# Clinal Variation in Reproductive Modes and Offspring Body Condition Across a Contact Zone of a Bimodal Viviparous Salamander

**DOI:** 10.1002/ece3.73223

**Published:** 2026-03-14

**Authors:** Clara Figueiredo‐Vázquez, Lucía Alarcón‐Ríos, Guillermo Velo‐Antón

**Affiliations:** ^1^ Departamento de Biologia da Faculdade de Ciências da Universidade do Porto Porto Portugal; ^2^ CIBIO/InBIO, Centro de Investigação em Biodiversidade e Recursos Genéticos da Universidade do Porto Instituto de Ciências Agrárias de Vairão Vairão Portugal; ^3^ BIOPOLIS Program in Genomics, Biodiversity and Land Planning CIBIO Vairão Portugal; ^4^ ECOEVO Lab, EE Forestal Universidad de Vigo Pontevedra Spain; ^5^ Departamento de Biología de Organismos y Sistemas, Área de Zoología Universidad de Oviedo Oviedo Spain

**Keywords:** bimodal reproduction, larviparity, life‐bearing, pueriparity, reproductive biology, *Salamandra salamandra*

## Abstract

Hybrid zones offer valuable insights into evolutionary processes. In species with bimodal reproduction, hybridization can produce transitional phenotypes that may pinpoint the evolutionary pathways connecting distinct reproductive modes. The fire salamander ( *Salamandra salamandra* ) is one of the two amphibians known to display bimodal viviparity, with populations exhibiting larviparity—birth of free‐living aquatic larvae—across most of its distribution, while pueriparity—birth of fully developed terrestrial metamorphs—is geographically restricted. These strategies differ in maternal investment, ontogeny, and offspring phenotype. Here, we investigate variation in reproductive modes and associated offspring body condition across a hybrid zone between two lineages, *S. s. gallaica* and *S. s. bernardezi,* that differ in reproductive (larviparous and pueriparous, respectively) and morphological traits (i.e., body size, head shape, and dorsal coloration). We aimed to characterize the spatial distribution of reproductive modes across the contact zone and explore potential fitness advantages of ‘pure’ and intermediate reproductive modes using differences in offspring body condition as an indirect proxy. We found a gradual and continuous shift in reproductive modes across the contact zone, with “pure” pueriparous mode in the north and larviparous in the south, and mixed reproductive modes in central populations, where hybrids appear to be viable and fertile. Offspring body condition varied significantly between reproductive modes, with offspring from mixed‐strategy females not only being viable but also exhibiting the highest body condition. This may result from reduced intra‐brood competition for matrotrophic resources, or alternatively, from increased sibling competition that favors offspring with higher performance. This system offers a unique opportunity to examine transitional viviparous forms in a natural context, providing an excellent model for understanding parity evolution, life‐history trade‐offs, and the evolutionary significance of reproductive shifts in vertebrates.

## Graphical abstract

We examined clinal gradients in reproductive modes and associated offspring body condition across a hybrid zone between two bimodal viviparous subspecies of *Salamandra salamandra* . We found a clear cline in reproductive modes, with pure modes at the extremes and mixed modes in central populations. Moreover, offspring body condition varied significantly across reproductive modes, with offspring from mixed clutches exhibiting the highest body condition.

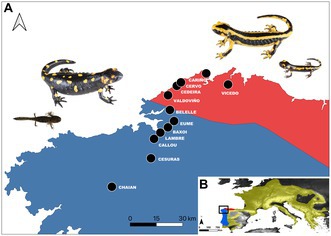

## 1 Introduction

Hybrid zones are regions where distinct evolutionary lineages meet and interbreed, producing offspring of mixed ancestry (Abbott et al. 2013; Barton and Hewitt 1985; Taylor et al. 2015; Abbott et al. 2016). The extent and outcome of hybridization depend on the strength of pre‐ and post‐zygotic reproductive barriers (Arnold 1992; Barton and De Cara 2009; Nosil et al. 2017; Ravinet et al. 2017). Prezygotic isolation includes premating barriers, such as sexual selection and assortative mating (Butlin and Smadja 2018; Van Schooten et al. 2020), and postmating barriers, such as genetic incompatibilities that prevent fertilization (Janoušek et al. 2012; Ravinet et al. 2017). Postzygotic isolation reduces hybrid viability and fitness (Butlin and Smadja 2018; Banker et al. 2022), thereby contributing to hybrid breakdown in areas of sympatry. Together, these barriers prevent lineage merging and promote divergence, potentially leading to complete reproductive isolation (Butlin and Smadja 2018; Dobzhansky 1937; Gompert and Buerkle 2016). Yet, closely related taxa hybridize more frequently than distant evolutionary taxa due to weaker pre‐ or post‐zygotic reproductive barriers (Dufresnes et al. 2023; Ambu et al. 2025).

Hybridization processes can also generate novel species (Salazar et al. 2010) and phenotypes (e.g., Seehausen 2004; Nichols et al. 2015; Lamichhaney et al. 2015; Akopyan et al. 2020), new functional traits (e.g., Rieseberg et al. 2003), the appearance of intermediate forms (Grant and Grant 1992; Whittington et al. 2022), and the reversal of a trait to the ancestral form (Stankowski and Streisfeld 2015; Velo‐Antón et al. 2023). When hybridizing lineages display polymorphic reproductive modes, hybridization can generate transitional forms that exhibit intermediate traits between the two strategies. Such hybridization events between lineages with polymorphic reproductive modes have been documented in vertebrates with bimodal reproduction: the scincid lizard (*Saiphos equalis*) and the common lizard (*Zootoca vivipara*) (Whittington et al. 2022). In these species, oviparous and viviparous populations breed along a hybrid zone, resulting in intermediate reproductive characteristics, such as advanced embryonic development at oviposition, a shortened incubation period, and reduced eggshell thickness (Smith and Shine 1997; Lindtke et al. 2010; Whittington et al. 2022). Yet, reproductive bimodality remains extremely rare in nature. Among vertebrates, strong evidence for reproductive bimodality has been documented (reviewed in Whittington et al. 2022) in only four reptile species: the already mentioned scincid lizard, *S. equalis* (Smith and Shine 1997), and the common lizard, *Z. vivipara* (Surget‐Groba et al. 2001), the Bougainville's skink, *Lerista bougainvillii* (Qualls and Shine 1998), and the brown‐banded water snake, *Helicops angulatus* (Rossman 1973); and in two amphibian urodele (salamanders and newts) sister species: the fire salamander, *Salamandra salamandra* (Dopazo and Alberch 1994; Velo‐Antón et al. 2007), and the North African fire salamander, *S. algira* (Donaire‐Barroso and Bogaerts 2001; Dinis and Velo‐Antón 2017).

Bimodal reproduction involves intraspecific variation in reproductive mode (e.g., oviparity, viviparity and transitional forms), resulting in differences in developmental trajectories (ontogeny), mother‐offspring relationships (maternal investment), and stages of development at birth (Buckley et al. 2007; Recknagel et al. 2021; Whittington et al. 2022). Evolutionary transitions in reproductive mode within reproductively bimodal species involve significant morphological (e.g., Stewart and Blackburn 2014; Greven 1998), physiological (e.g., Recknagel et al. 2021), life‐history (e.g., Buckley et al. 2007; Velo‐Antón et al. 2015; Recknagel and Elmer 2019), behavioral (e.g., parental care: Kupfer et al. 2006), ecological (Horreo et al. 2021) and genetic (Recknagel et al. 2021) changes. These exceptional systems thus provide a unique opportunity to study reproductive transitions, as gene flow may promote the emergence of intermediate forms that might otherwise be lost during the speciation process (Roitberg et al. 2013; Blackburn 2015).

The two bimodally reproductive urodele species present two modes of viviparous (live‐bearing) reproduction: an ancestral larviparous mode, in which offspring undergo part of their development as free‐living aquatic larvae, and the derived pueriparous mode (sensu Greven 2003), in which fully developed terrestrial metamorphs are born (Buckley et al. 2007; Dopazo and Korenblum 2000; Velo‐Antón et al. 2015). Unlike in squamates and two strict pueriparous salamander species, the alpine salamanders *S. atra* and *S. lanzai*, pueriparity in *S. salamandra* does not involve prolonged egg or larval retention (Buckley et al. 2007). Instead, pueriparity is associated with fixed changes in the rate and sequence (heterochrony) of intrauterine development coupled with matrotrophy, extending nutritional supplementation of the developing offspring beyond what can be achieved by lecithotrophy, or nutrition derived from egg yolk (Buckley et al. 2007). Specifically, after depleting the egg‐yolk reserves, the hatched embryos feed actively within the female reproductive tract on arrested eggs (oophagy) and less developed siblings (adelphophagy), whereas in larviparous individuals, active feeding begins only after the larvae enter the aquatic environment (Buckley et al. 2007; Alarcón‐Ríos and Velo‐Antón 2024). Consequently, life‐history traits related to reproductive outcomes, such as newborn weight, size, as well as body condition, differ between both reproductive modes (Velo‐Antón et al. 2015). Evidence from natural populations suggests that transitions between these reproductive modes may not always be discrete. Previous studies have reported intermediate reproductive forms in *S. salamandra* , where females produce both aquatic larvae and terrestrial juveniles within the same clutch (Galán 2007; Uotila et al. 2013; Velo‐Antón et al. 2015; Mulder et al. 2022). In addition to these outcomes from natural hybrid zones between larviparous and pueriparous lineages, there is also evidence of F1 hybrids, and the reversal of the parity mode, resulting from a crossing between a pueriparous female and a larviparous male (Velo‐Antón et al. 2023). However, the reproductive outcomes of these intermediate forms in natural populations remain largely understudied.

By studying these transitions in a natural contact zone, we aim to evaluate the existence of intermediate reproductive modes, their role in the evolutionary shift from larviparity to pueriparity in *S. salamandra* , and whether transitional forms are associated with distinct reproductive outcomes—particularly offspring body condition—that could indicate a fitness advantage or constraint. First, we aim to characterize the reproductive mode of *S. salamandra* females across a hybrid zone between two evolutionary lineages, the larviparous *S. s. gallaica* and the pueriparous *S. s. bernardezi*. Second, we aim to assess the eco‐evolutionary implications of intermediate modes in relation to the pure modes. This hybrid zone, which includes the area where both reproductive modes are expected to meet (Galán 2007), has been spatially and genetically characterized, showing high levels of admixture and introgression, with patterns consistent with extensive gene flow and the absence of reproductive barriers (Velo‐Antón et al. 2021). The absence of environmental or pre‐zygotic barriers (especially at the syntopic zone) and landscape barriers to gene flow (Velo‐Antón et al. 2021) makes this contact zone ideal for characterizing the reproductive mode and its transitions at the zone of syntopy.

We sampled pregnant females along a geographic transect across the contact zone between *S. s. gallaica* and *S. s. bernardezi* and characterized the birth developmental stage of newborns to: (1) delineate the geographic distribution of both reproductive modes and possible intermediate modes along the transect; and (2) examine variation in offspring body condition across reproductive modes. We hypothesized the following: (H1) the extremes of the transect consist of “pure” reproductive modes—pueriparous in the north and larviparous in the south—without the presence of mixed modes, while mixed modes are found in contact zones, coinciding with the genetic characterization within this hybrid zone (NG in Velo‐Antón et al. 2021). (H2) Populations located at the centre of the reproductive transect will exhibit an approximately equal number of pueriparous and larviparous clutches, with a gradual shift toward dominance of larviparous clutches to the south and pueriparous clutches to the north. Finally, due to the occurrence of additional food supply (matrotrophy) in the pueriparous strategy (Buckley et al. 2007; Alarcón‐Ríos and Velo‐Antón 2024), we also expect (H3) higher body condition in both pueriparous and mixed modes at (H3.1) the individual offspring level (comparing newborn developmental stages) and (H3.2) the female level (comparing offspring from females with different reproductive modes), and (H3.3) higher variance in offspring body condition from matrotrophic mixed and pueriparous females than from exclusively lecithotrophic larviparous females.

## 2 Material and Methods

### 2.1 Study System

*Salamandra salamandra gallaica* and *S. s. bernardezi* diverged during the Pleistocene in the northern Iberian Peninsula (Burgon et al. 2021; Velo‐Antón et al. 2021; Mulder et al. 2022; Gippner et al. 2024). In *S. s. bernardezi*, pueriparity arose during the Pleistocene (0.26–1.78 Mya) in the Cantabrian Mountains (NW Spain), while two independent origins of pueriparity occurred during the Holocene in insular populations of *S. s. gallaica* (Velo‐Antón et al. 2007; Velo‐Antón et al. 2012; Mulder et al. 2022). Pueriparity in *S. s. bernardezi*, as well as in other pueriparous lineages within *Salamandra* (with the exception of the insular *S. s. gallaica*) and *Lyciasalamandra*, likely evolved in regions with steep topography and karstic lithology through local adaptive processes associated with the scarcity or absence of surface water required in the larviparous strategy (Dinis et al. 2025). During subsequent warm and cold periods of Pleistocene climatic oscillations, both pueriparous *S. s. bernardezi* and larviparous *S. s. gallaica* might have experienced cycles of isolation and expansion in response to unfavorable and favorable conditions, respectively (Dinis et al. 2025). These range shifts, combined with the absence of ecological divergence mediated by climate or land cover between parity modes (Dinis et al. 2025), facilitated secondary contact between the lineages (García‐París et al. 2003). This contact shows evidence of past mitochondrial introgression (Lourenço et al. 2019) and established the present contact zone in northern Galicia, NW Spain (Velo‐Antón et al. 2021). In addition to reproductive mode, these lineages also differ in multiple phenotypic traits including body size (Velo‐Antón et al. 2015), head shape (Alarcón‐Ríos, Nicieza, Kaliontzopoulou, et al. 2020), and dorsal coloration (Alarcón‐Ríos et al. 2024). Previous studies have identified ecological divergence between reproductive modes, primarily related to topography (slope) and lithology (karst), with no significant differences in other climatic variables, such as mean temperature (Dinis et al. 2025). A relatively high degree of niche overlap exists between pueriparous and larviparous salamanders, indicating that most habitats suitable for pueriparity can also support larviparity when water bodies are present (Dinis et al. 2025). Therefore, these shared environments provide a suitable framework for comparing reproductive modes.

### 2.2 Study Site and Sampling

**FIGURE 1:**
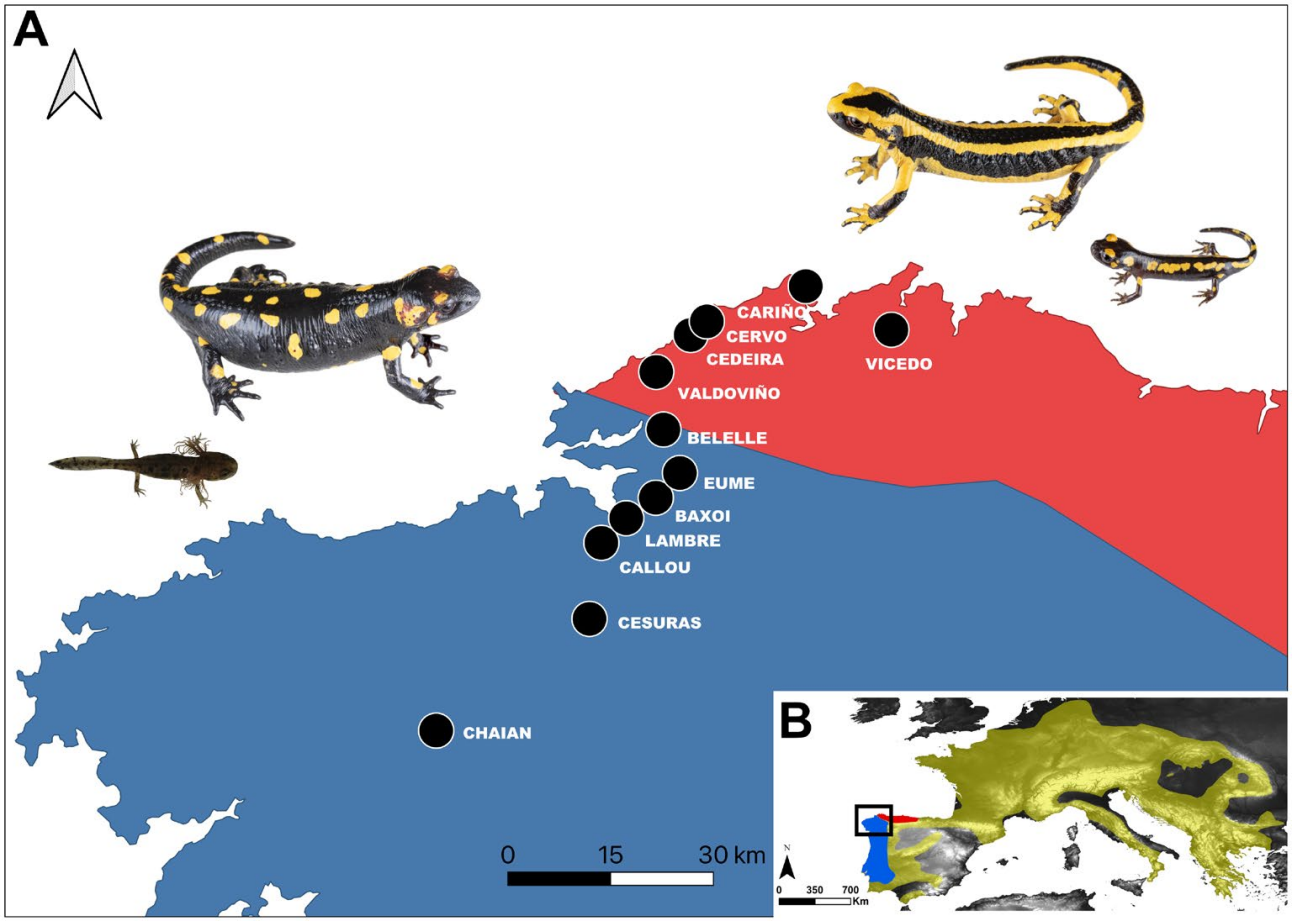
(A) Approximate contact zone between the two *Salamandra salamandra* subspecies based on Velo‐Antón et al. (2021). The distribution ranges of *S. s. gallaica* and *S. s. bernardezi* are shaded in blue and red, respectively. Representative images of *Salamandra salamandra gallaica* and *S. s. bernardezi*, along with their respective larvae and metamorphs are represented (credits: Max Benito and Jose Vieira, ExSitu Project). (B) Distribution of *S. salamandra* depicted in yellow with *S. s. gallaica* and *S. s. bernardezi* ranges highlighted in blue and red, respectively. The studied contact zone is indicated by a black square.

We studied an environmentally and genetically characterized contact zone between *S. s. gallaica* and *S. s. bernardezi* subspecies, located in northwestern Galicia, NW Spain, at the westernmost range of *S. s. bernardezi* (Velo‐Antón et al. 2021) (Figure 1). We established a transect along a north–south axis, spanning approximately 105.5 km (measured as a straight line between the northernmost and southernmost populations—Figure 1A). This transect encompasses populations identified as putatively pure *S. s. bernardezi* and *S. s. gallaica*, as well as the contact zone where relatively high levels of gene flow, hybridization, and intermixed phenotypes between both lineages were previously found (Velo‐Antón et al. 2021). Genetic differentiation across this contact zone is mainly predicted by isolation by distance, emphasizing the absence of climatic or topographical barriers, including the main river found along the transect (Figueiredo‐Vázquez et al. 2021; Velo‐Antón et al. 2021). Morphologically, *S. s. gallaica* individuals are characterized by their larger body size (up to 250 mm), blotched/spotted dorsal coloration, and a relatively pointed snout. In contrast, *S. s. bernardezi* individuals are smaller (up to 180 mm), exhibit striped dorsal patterns, and have rounded snouts (Figure 1A) (Velo‐Antón and Buckley 2015; Alarcón‐Ríos, Nicieza, Kaliontzopoulou, et al. 2020; Alarcón‐Ríos et al. 2024).

We sampled 16 populations during rainy nights between spring 2022 and autumn 2024, collecting 116 potentially gravid females (Table 1; Figure 1). Additionally, we included data from one female previously sampled near the northernmost locality (Vicedo; Mulder et al. 2022). Gravidity was assessed based on the size and shape of the lower abdomen, where the uterus is located (Buckley et al. 2007; Velo‐Antón et al. 2015; Alarcón‐Ríos, Nicieza, Lourenço, and Velo‐Antón 2020). As an indirect sign of the population's reproductive mode, we also opportunistically checked for the presence of larvae in water bodies (e.g., streams, ponds, fountains), without following a systematic approach or predefined study design.

**TABLE 1:** Sampling effort: Number of potentially gravid females (*N*) captured in each population, listed from north to south. Population acronyms are provided in parentheses. The number of recorded births (*N* births) and the observed reproductive strategy for each population are also shown. The percentage of females exhibiting each reproductive strategy (*fem_mode*) and the summary statistics for body condition (SMI) are shown for each population.

Population	*N*	*N* births	Reproductive mode	fem_mode			SMI	
				% larv.	% puer.	% mixed	Mean ± SD	Median
CARIÑO (CAR)	19	1	Pueriparity^a^	0	100	0	0.24 (± 0.02)	0.24
O VICEDO (VIC)^b^	1	1	Pueriparity^a^	0	100	0	NA	NA
MIRADOURO DE LIMO (LIM)	5	0	NA^a^	NA	NA	NA	NA	NA
GARITA point B (GAR B)	4	0	NA^a^	NA	NA	NA	NA	NA
GARITA DA HERBEIRA (GAR)	10	0	NA^a^	NA	NA	NA	NA	NA
SAN ANDRÉS DE TEIXIDO	12	0	NA	NA	NA	NA	NA	NA
CERVO (CER)	15	2	Mixed	0	0	100	0.38 (± 0.12)	0.36
CEDEIRA (CED)	5	2	Mixed	0	50	50	0.38 (± 0.12)	0.34
VALDOVIÑO (VAL)	13	4	Mixed	0	25	75	0.3 (± 0.05)	0.3
BELELLE (BEL)	2	2	Mixed	50	50	0	0.28 (± 0.1)	0.24
EUME (EUM)	3	3	Mixed	0	0	100	0.26 (± 0.09)	0.24
BAXOI (BAX)	5	4	Mixed	25	75	0	0.25 (± 0.04)	0.24
LAMBRE (LAM)	6	4	Mixed	0	0	100	0.34 (± 0.1)	0.31
CALLOU (CAL)	4	4	Larviparity	75	25	0	0.28 (± 0.07)	0.26
CESURAS (CES)	4	2	Larviparity	100	0	0	0.24 (± 0.04)	0.24
CHAIAN (CHA)	9	3	Larviparity	100	0	0	0.23 (± 0.07)	0.22

^a^ No larvae were observed during opportunistic visual surveys of water bodies.

^b^ Data for the O Vicedo population were reused from a different study with unrelated research objectives.

### 2.3 Animal Maintenance

We maintained all females under standardized outdoor conditions (Velo‐Antón et al. 2015; Alarcón‐Ríos and Velo‐Antón 2024). We collected and transported the females to an outdoor enclosure (University of Vigo, Spain), with water availability and high levels of humidity and shade, providing climatic conditions close to what they would experience in their natural environment, and hence suitable for fire salamanders, which naturally occur in the surrounding area. Nevertheless, we acknowledge that fine‐scale differences in climate and breeding conditions may exist between parity modes in nature, although these remain understudied (Dinis et al. 2025).

We placed the salamanders in individual terraria (36 × 25 × 17 cm; L × W × H), filled with coconut fiber as substrate, and equipped with branches, bricks, or bark as shelter and hiding places, and water‐filled plastic boxes (3–4 cm deep) for potential larviposition. We fed them with earthworms ad libitum and checked them twice a week for possible parturitions of aquatic larvae and/or terrestrial metamorphs. We maintained females for up to 3 weeks after the birth of their last offspring. If there was no parturition, we maintained them until the end of each activity season. All females and offspring were released at their exact place of capture.

Salamanders were captured and processed under collection permits provided by regional governments (Xunta de Galicia, Ref. EB‐049/2022; EB‐039/2023; EB‐091/2024). All applicable national and institutional guidelines for the care and use of animals were followed.

### 2.4 Data Collection

We measured the weight and body length (SVL; from snout to cloaca) of females upon arrival at the laboratory, prior to parturition. When newborns were detected in the terraria, we counted the number of newborn juveniles and/or larvae for each female and measured the weight and total length (TL; from snout to tail tip) of each newly born individual. We used a scale and a digital caliper to measure to the nearest 0.01 g and mm, respectively, for all measurements. We also took dorsal pictures of each female and newborn, including a 1 mm grid paper for scale. We discarded measurements from those individuals for whom it was difficult to take them (e.g., dead highly degraded larvae). For newborns for which direct length measurements were not possible, length was subsequently measured from photographs using the software Fiji‐ImageJ (Schindelin et al. 2012). We did not obtain quantitative data on body mass or total length of the newborns from the O Vicedo population, as this birth sample was collected for a previous study with different objectives (Mulder et al. 2022).

### 2.5 Classification of Newborn Character Stages

**FIGURE 2:**
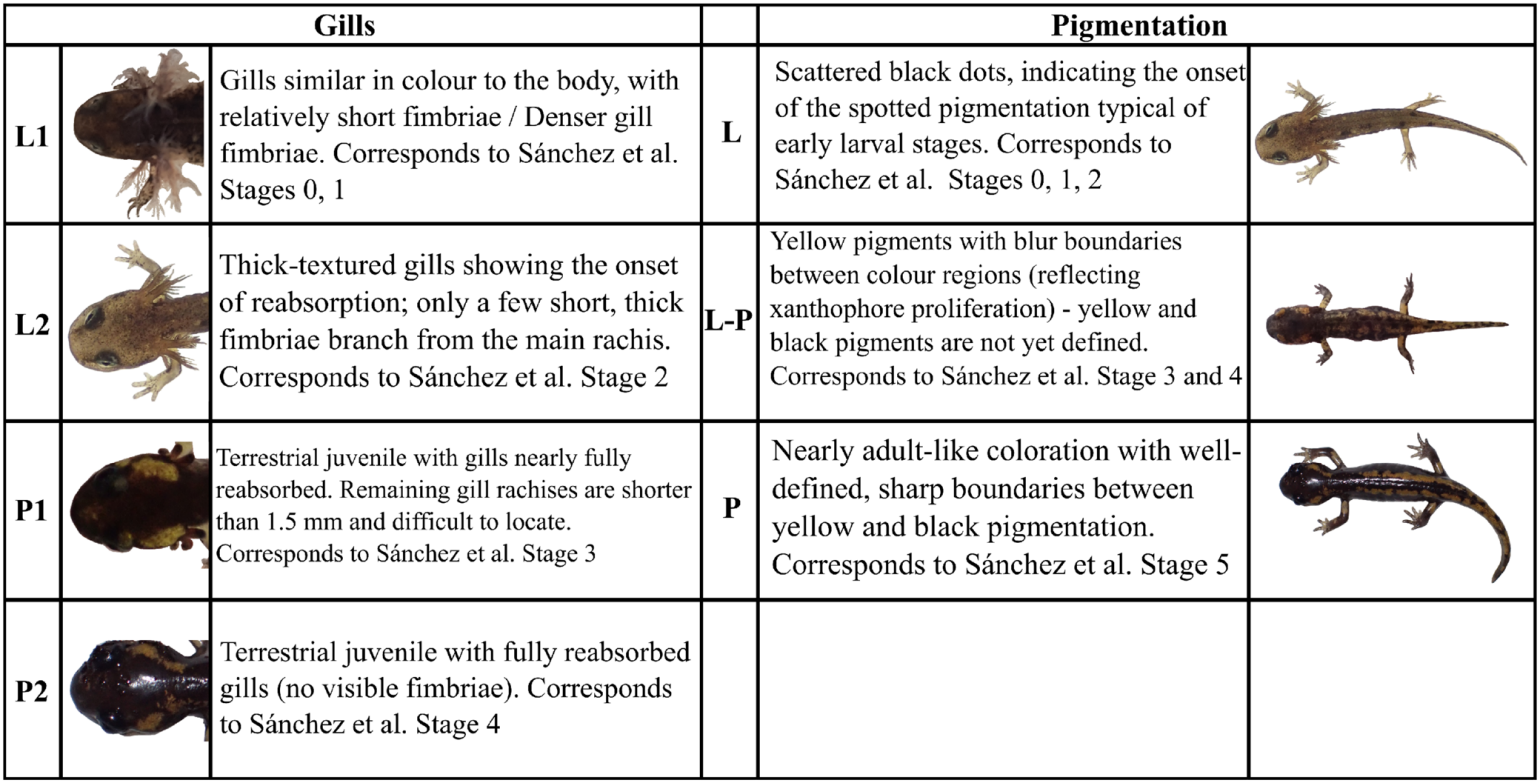
Main characteristics of development stages for gill morphology and coloration, and correspondence to the developmental stages from Sanchez et al. (2018). A representative image for each stage is included.

**FIGURE 3:**
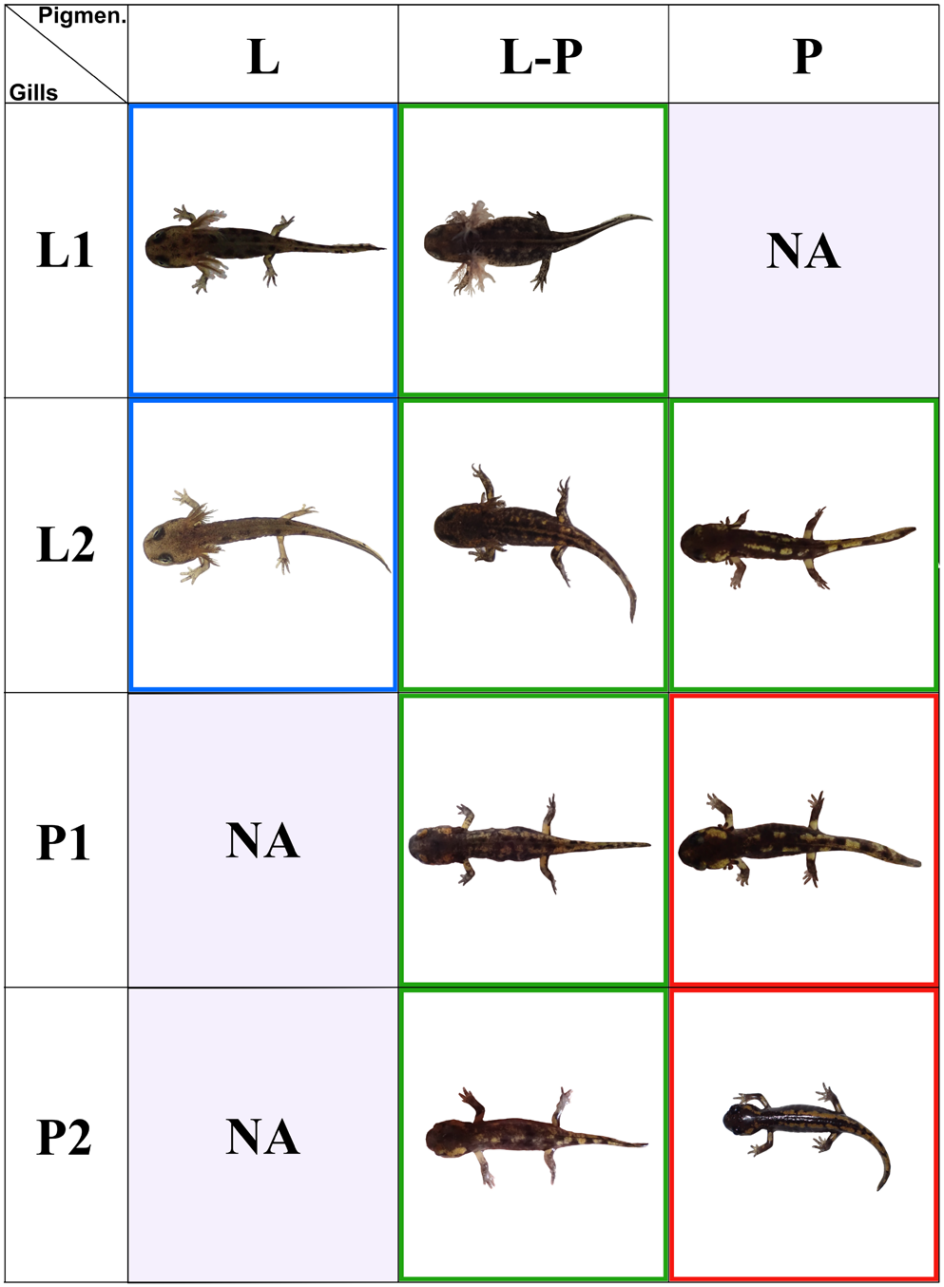
This figure illustrates the possible combinations of developmental stages for gill morphology (L1, L2, P1 and P2) and pigmentation (L, LP, P). Combinations with no available records are labeled as ‘NA’. Larvae, mixed and pueriparous phenotypes are indicated with blue, green and red squares, respectively.

To determine the developmental stage of the progeny, we classified each individual based on specific characteristics of gill morphology and pigmentation, which reflect the ontogenetic changes occurring during larval development, as originally described for larviparous individuals (Sanchez et al. 2018). The character stages (Figures 2 and 3) are qualitative and based on a modified version of supporting information 1 from Sanchez et al. (2018). This modification aims to reduce noise in our dataset, which shows high variability in character stages due to sampling within a hybrid zone and the presence of broods containing offspring at different developmental stages (e.g., pueriparous and mixed births), as we are studying lineages with heterochronic changes in ontogeny (Buckley et al. 2007). To accommodate this variability—without creating too many artificial categories and considering the limited quality of some images—we adopted a simpler classification scheme that remains informative for characterizing the contact zone and inferring reproductive modes. We propose three stages for pigmentation (L, L‐P, and P), and four stages for gill development (L1, L2, P1, and P2), each summarizing distinct phases of larval ontogeny (Figures 2 and 3). Unlike Sanchez et al. (2018), we did not include tail development in our staging system, as capturing high‐quality images of the tail requires multiple viewing angles (from above and from the side). However, since our developmental traits are not independent (Chi‐square *p*‐value < 2.2e‐16; see results), excluding tail traits is unlikely to affect our overall interpretation of the hybrid zone.

At the first stage of pigmentation (L) the color pattern is characteristic of aquatic individuals. Individuals in this phase do not show a defined black or yellow pattern. Instead, early in their ontogeny, they display a dark overall coloration with visible melanophores, which transition into a lighter gray pattern with fewer visible spots. In the intermediate stage (L‐P), the boundaries between the color regions are not sharp, and yellow starts to emerge, while at the final stage (P), individuals display a metamorphic coloration characterized by distinct, sharp boundaries between yellow and black spots (Figures 2 and 3).

Two (L1 and L2) of the four stages for gill morphology development correspond to larviparous stage, while the other two (P1 and P2) correspond to the pueriparous stage. In the first stage (L1), gills are characterized by dense, elongated fimbriae. In stage L2, gills begin to be reabsorbed and develop a thicker texture, with only a few short, thick fimbriae branching from them. By stage P1, the individual has gills almost entirely reabsorbed, and in stage P2, the gills are completely absent (Figures 2 and 3).

Each individual was assessed independently by three observers to mitigate potential biases in visual inspection. In the few cases where consensus was not initially reached, photographs were reviewed and discussed until agreement was achieved.

### 2.6 Reproductive Mode Characterization

We employed a three‐level hierarchical classification framework based on newborn developmental traits (see previous section), distinguishing offspring developmental character stages at the individual level, female (clutch) reproductive modes, and population‐level patterns.

First, at the individual level (hereafter offspring character stage), individuals were classified as larvae when the pigmentation stage corresponded to that of early larval stage (L), and gill development fell within L1 or L2, which are considered to be fully functional in water. Terrestrial juveniles were identified by pigmentation at stage P and gill development at stage P1 (absence of fimbriae) or P2. Intermediate individuals were identified based on the presence of developmental characteristics that were transitional between aquatic and terrestrial states. These included individuals classified as L‐P for pigmentation and any gill developmental stage, as well as P pigmented with L2 gills. Mixed individuals were those displaying asynchronous development, with notable differences between gill and pigmentation stages (e.g., gills at stage L2 and pigmentation at stage P). Examples of all developmental stages are presented in Figure 3.

Second, the reproductive mode of each clutch (i.e., female) was categorized according to the phenotypic traits of the newborns it contained. Larviparous births were defined as those containing only aquatic larvae (gill/pigmentation: L1/L or L2/L). Pueriparous births were defined as those containing exclusively terrestrial juveniles (gill/pigmentation: P1/P or P2/P). Mixed births were defined as those that met at least one of the following criteria: (1) individuals exhibiting asynchronous development between gill and pigmentation stages; (2) siblings at markedly different developmental stages, such as the coexistence of aquatic larvae and terrestrial juveniles within the same clutch (Figure 3).

Finally, the reproductive mode of the population considers all births from females within each location. Accordingly, we classified as larviparous or pueriparous those populations consisting exclusively of larviparous or pueriparous births, respectively. We identified as mixed populations those with co‐occurring larviparous and pueriparous births, as well as those containing mixed births.

**FIGURE 4:**
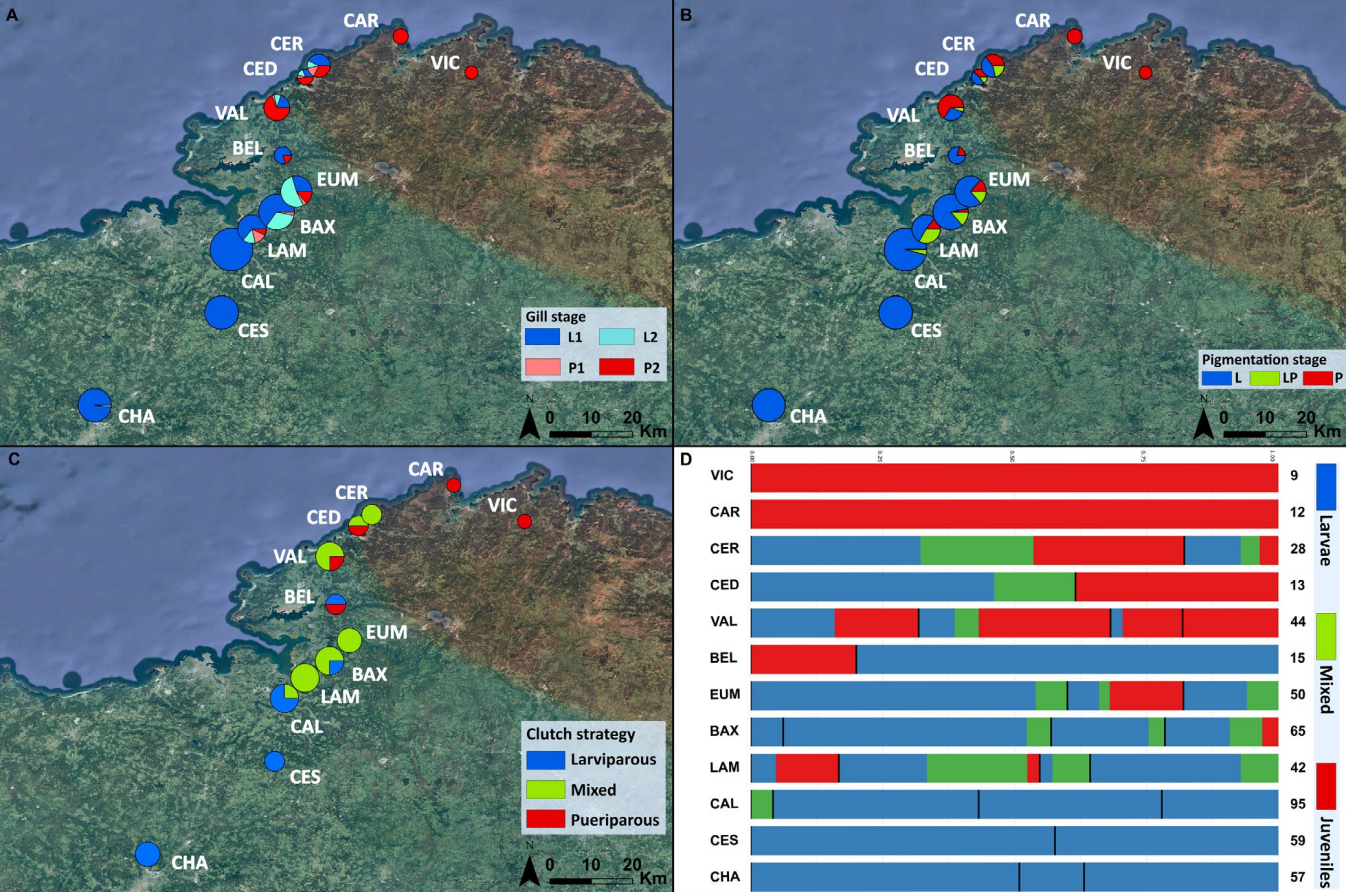
Pie charts showing the frequencies of different developmental stages for (A) gills and (B) pigmentation of the offspring in each population. Pie chart size is proportional to the number of offspring sampled per population. (C) Proportions of reproductive modes at the clutch level, with pie chart size proportional to the number of clutches sampled per population. Developmental character stages and reproductive modes are color‐coded according to the legends in each panel. (D) Proportions of the three offspring developmental character stages modes—larvae (blue), mixed (green), and juveniles (red)—based on individual‐level classification across different populations arranged from north to south. Each bar represents a single population and is divided by clutch (individual female), indicated by horizontal black lines. Sample sizes (*N*) for each population are indicated. All population names are indicated by their acronyms.

To assess the extent of reproductive mode mixing at the clutch (female) and population levels, we calculated the proportion of larval, juvenile, and mixed offspring within (i) each clutch and (ii) each population, as well as the proportion of larviparous, pueriparous, and mixed clutches per population (Figure 4C,D).

### 2.7 Correspondence Analyses of Offspring Character Stages

We performed a Correspondence Analyses (CA) using the *FactoMineR* R package (v. 2.8; Lê et al. 2008) on categorical data to explore patterns of association among the different offspring character stage patterns observed at different localities. CA was applied to population‐level contingency tables, in which rows corresponded to localities and columns to character stage categories, using the frequencies of offspring exhibiting each gill and pigmentation stage to generate a distance matrix. This matrix was visualized in a biplot, allowing associations among populations and character stage categories to be interpreted based on their relative proximity in the ordination space (Glynn 2014). To account for mixed individuals and heterochrony between gill and pigmentation developmental stages, we performed a Correspondence Analysis that considered the levels of each variable independently, as well as an additional analysis based on the combined levels of both variables.

Finally, we performed a Pearson's Chi‐squared test of independence to statistically assess the association between the variables gills and pigmentation.

### 2.8 Body Condition

To establish an allometric relationship between body mass and size, we calculated the Scaled Mass Index (SMI) as a proxy for body condition (Peig and Green 2009). SMI provides a standardized measure of body mass relative to length, serving as a robust indicator of offspring condition (Peig and Green 2009). In amphibians, body condition has been shown to correlate with energy reserves (MacCracken and Stebbings 2012), serving as a useful surrogate for offspring fitness and survival probability. Each individual's body length was standardized using the mean Total Length (TL) of the entire offspring dataset (TL_0_), adjusting the observed mass to the value it would have at this standardized length. This approach enables direct comparisons of body condition across different clutches and populations (Peig and Green 2009; Alarcón‐Ríos and Velo‐Antón 2024). Offspring with missing data for total length, mass, or both were excluded from the analysis, resulting in the exclusion of 7.5% of individuals (see also Results).

We tested for a correlation between weight and total length (*r* = 0.813, *p* = 0.001). Given this strong correlation, all model analyses were conducted using the SMI. We calculated descriptive statistics (arithmetic mean ± standard deviation, median, and range) for SMI for each reproductive mode at three hierarchical classification levels: (1) offspring developmental character stage, (2) female (clutch) reproductive mode, and (3) population‐level reproductive mode (Table A1). Accordingly, individual offspring were grouped into three categories (larviparous, pueriparous, mixed) based on their own developmental character stage, the reproductive mode of their mother, and the reproductive composition of the population in which they were born (Table A1).

### 2.9 Body Condition Variation Across Reproductive Modes

To examine variation in offspring body condition across reproductive modes, we fitted a linear mixed‐effects model (*model.1*) using offspring log‐transformed SMI (*logsmi*) as the response variable. Fixed effects included scaled maternal snout–vent length (*fsvl_scaled*), its quadratic term (*fsvl_scaled_sq*) to account for potential non‐linear effects, scaled clutch size (*nclutch_scaled*), offspring character stage (*offspring_stage*), and maternal reproductive strategy (*fem_mode*). To account for the nested structure of the data and repeated measures within females and populations, we included random intercepts for population and for female identity nested within population (1 | *pop/female*). The model was fitted using the lme() function from the nlme package (Pinheiro et al. 2025) in R version 4.1.2 (R Core Team 2021), with parameters estimated via restricted maximum likelihood (REML). To allow for potential differences in residual variance across maternal reproductive strategies, we specified a varIdent() variance structure by *fem_mode*. This approach enabled us to test whether offspring resulting from different maternal reproductive modes differed not only in mean body condition, but also in variability in SMI, after accounting for fixed and random effects.

**FIGURE A1:**
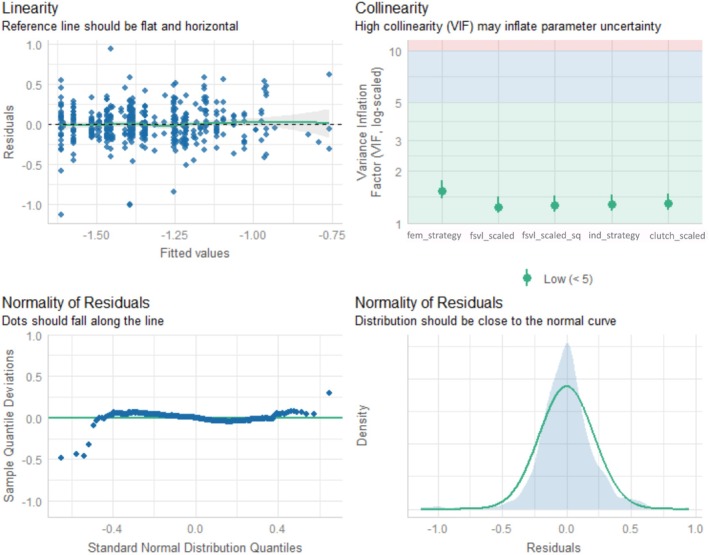
Graphical model diagnostics for the heteroscedastic linear mixed‐effects model (*model.1*). Plots illustrate the evaluation of key model assumptions: Plots show (top left) residuals versus fitted values to assess linearity, (top right) variance inflation factors (VIFs) for fixed effects to assess collinearity, (bottom left) Q–Q plot of standardized residuals to assess normality, and (bottom right) kernel density plot of residuals compared to a normal distribution.

To test for pairwise differences in offspring body condition among reproductive modes, we conducted post hoc comparisons of estimated marginal means (EMMs) with Tukey adjustment for multiple comparisons, using the *emmeans* package (Lenth et al. 2025). The Intraclass Correlation Coefficient (ICC) was used to estimate the proportion of variance attributable to random effects (female identity and/or population). Model validation was performed using the check_model() function from the performance package (Lüdecke et al. 2021). The complete validation procedure and corresponding plots are provided in Text A1 and Figure A1.

## 3 Results

### 3.1 Reproductive Mode Characterization

We obtained birth data for 549 newborn individuals from 33 females across 12 populations (including one female from a previous study). Due to inherent uncertainty in confirming pregnancy status at the time of collection, some sampling attempts were unsuccessful or involved non‐pregnant females (Table 1 and Table A2). Across all populations, 32 of the 117 females produced clutches, corresponding to an overall success rate of 27%. However, success rates differed among reproductive modes, averaging 10% in pueriparous, 53% in larviparous, and 43% in mixed populations (Table 1).

We found a clear cline in reproductive modes from the north to the south along our sampling transect. Specifically, the northernmost populations, Cariño and O Vicedo, were fully pueriparous, as all females gave birth to fully metamorphosed terrestrial juveniles with either P1/P or P2/P gills and pigmentation stages (Table 1; Figure 4; Table A2). Moreover, no larvae were observed during the opportunistic visual surveys of water bodies in populations presumed to be pueriparous (Cariño, Vicedo). On the other extreme, populations at the southernmost region of our transect, Cesuras and Chaián, resulted in fully larviparous clutches, with all individuals exhibiting L1/L gills and pigmentation character stages (Table 1; Figure 4; Table A2).

Central populations along the cline (Cervo, Cedeira, Valdoviño, Belelle, Eume, Baxoi, Lambre, and Callou) exhibited a mixed reproductive mode. This included a predominance of mixed clutches (e.g., Cervo, Eume, Baxoi, and Lambre); the co‐occurrence of mixed clutches with either pure pueriparous (Cedeira and Valdoviño) or pure larviparous (Callou) births; or the simultaneous presence of both larviparous and pueriparous clutches within the same population (Belelle) (Table 1; Figure 4; Table A2).

### 3.2 Individual Character Stage Patterns

We measured gill and pigmentation developmental stages in 467 individuals from 32 females across 12 populations. We excluded the Belelle population due to ambiguous birth compositions that could lead to misclassification. Specifically, we were unable to obtain character stage information for seven newborns—presumably metamorphs—out of 19 individuals in female BEL_20F, where the remaining individuals were larvae. The second birth (BEL_21F) consisted of three metamorphs.

**FIGURE 5:**
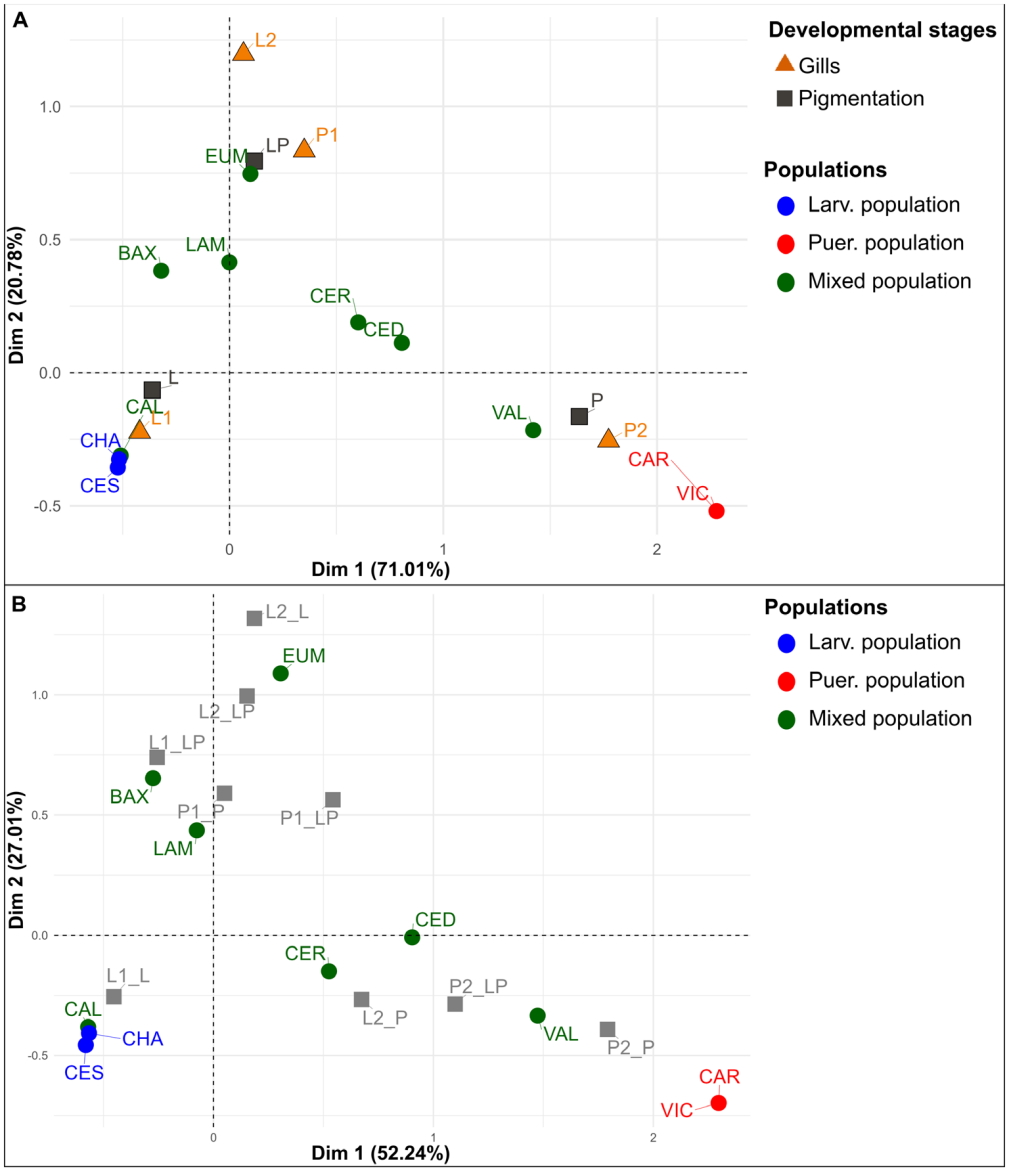
(A) Symmetric plot of the first two dimensions from the Correspondence Analysis (CA). Populations are represented by circles, colored according to their reproductive mode (blue: Larviparous; green: Mixed; red: Pueriparous). Gill developmental stages (L1, L2, P1, P2) are shown as orange triangles, and pigmentation stages (L, LP, P) as dark gray squares. (B) Symmetric plot of the first two dimensions from the CA based on the combination of gill and pigmentation developmental stages. Populations are shown as circles colored according to reproductive mode (blue: Larviparous; green: Mixed; red: Pueriparous), whereas combinations of developmental stages are shown as squares.

**FIGURE A2:**
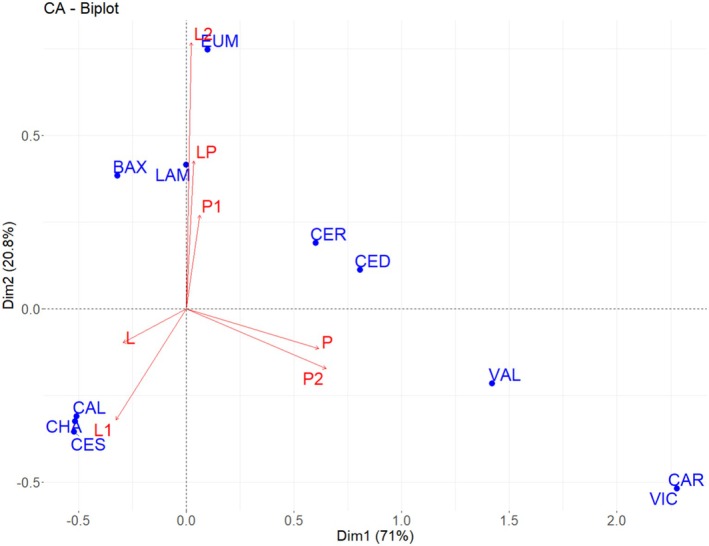
Asymmetric biplot showing the contribution of gill and pigmentation developmental stages (red arrows) to the variation explained by the first two dimensions. Dimension 1 (71%) distinguishes larviparous from pueriparous populations, while Dimension 2 (20.8%) reflects variation within mixed populations. The distance of each variable point (red arrow) from the origin reflects its contribution to factor map, and the angle of each arrow relative to the axes indicates the strength and direction of its association with each dimension. Blue points represent the location of populations along both axis of variation.

For the Correspondence Analysis considering gills and pigmentation stages independently, we retained the first two factorial axes, which together explained 91.8% of the total variance, accounting for 71.01% and 20.78%, respectively (Figure 5A). Dimension 1 captured the main contrast between larviparous and pueriparous populations. Larviparous populations (e.g., Chaián, Cesuras) and some mixed ones (e.g., Callou) were positioned on the left, while pueriparous populations (e.g., Cariño, O Vicedo) were on the right, following a south–north axis. This axis was largely defined by early larval stages (L1, L) and late metamorphic character stages (P2, P). The asymmetric biplot (Figure A2), which illustrates the contribution of character stage categories to the two main dimensions, shows that Dimension 1 is associated with gill stages (L1 and P2), though it is more strongly influenced by pigmentation stages L and P. The gill stage L1 contributes to both dimensions, as indicated by its intermediate position between the axes; stage P2 also contributed to both, but more prominently to Dimension 1 (larviparous vs. pueriparous) than to Dimension 2 (pure vs. mixed). A second dimension, which explained 20.78% of the variation, separates the mixed populations and intermediate developmental stages (Figure A2). It was mainly influenced by intermediate gill (L2, P1) and pigmentation stages (LP) (Figure A2), which positioned mixed populations centrally, reflecting their transitional character profiles.

The results of the Pearson's Chi‐squared test were statistically significant (*p* < 2.2e‐16), indicating an association between the gills and pigmentation developmental stages. For the Correspondence Analysis based on population‐level frequencies of combined gill and pigmentation character stage categories, we retained the first two factorial axes, which together explained 79.25% of the total variance (52.24% and 27.01%, respectively; Figure 5B). Similar to the Correspondence Analyses that consider gill and pigmentation stages independently, two primary axes of variation emerged. Along Dimension 1, larviparous populations (Chaián, Cesuras) are positioned on the left, while pueriparous populations (Cariño and Vicedo) are located on the right. Intermediate mixed populations occupy central positions, indicating a clear separation between larviparous and pueriparous populations. Dimension 2 primarily differentiates the mixed populations. The spatial distribution of developmental character stages supports these two main axes of variation. Early larviparous stages (L1_L) cluster on the left along Dimension 1, while later pueriparous stages (P2_P) are positioned on the right. Character stage combinations characteristic of mixed offspring (L2_P, P2_LP) appear near the centre of Dimension 1. Dimension 2 separates intermediate mixed stages (L1_LP, L2_LP, P1_LP), while larval (L2_L) and metamorphic (P1_P) stages also show strong association with this axis.

### 3.3 Body Condition Variation Across Reproductive Modes

**FIGURE 6:**
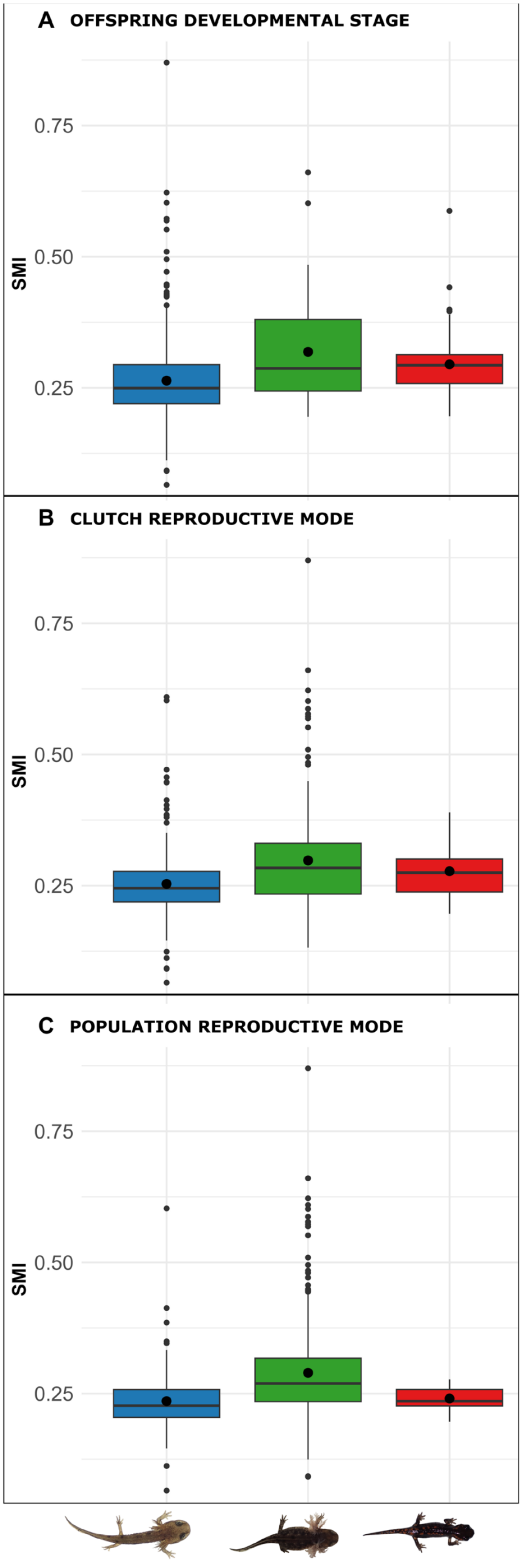
Boxplots showing differences in Scale Mass Index (SMI) across reproductive strategies, categorized by (A) offspring developmental character stage (B) female (clutch) reproductive mode and (C) population reproductive mode. Illustrations representing the developmental categories are shown below the panels. In all boxplots, black horizontal lines represent the median, and black circles within the boxes indicate the mean. Each box spans from the first quartile (Q1) to the third quartile (Q3), representing the interquartile range (IQR). Whiskers extend to the smallest and largest values within 1.5 × IQR from the hinges (Q1 and Q3). Values outside this range are plotted as individual outlier points.

**FIGURE A3:**
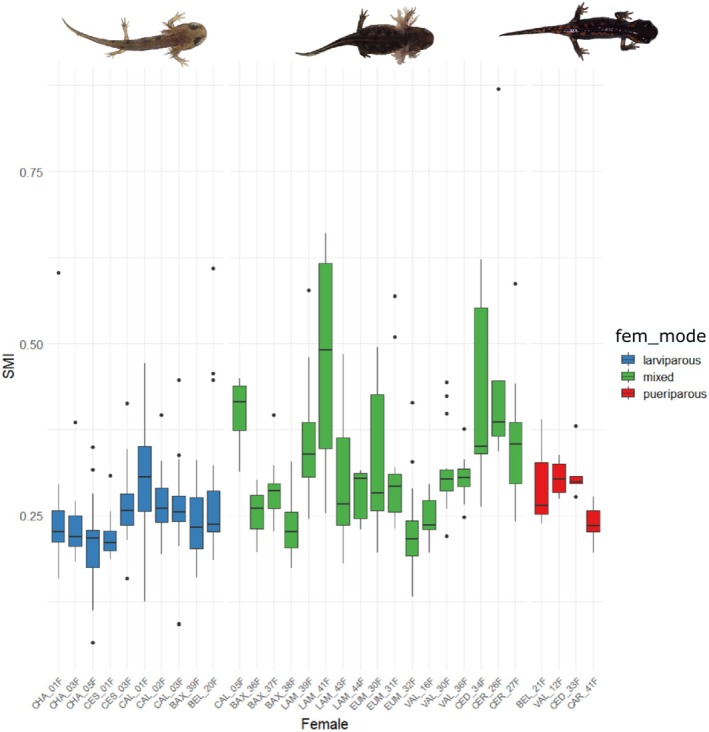
Boxplot showing offspring body condition (Scale Mass Index, SMI) per female. Females are grouped and colored according to their reproductive mode: Larviparous (blue), mixed (green), and pueriparous (red). Within each reproductive mode, females are ordered from south to north. Medians are represented as black horizontal lines, boxes indicate the interquartile range (IQR), and black dots represent outliers.

Newborn SMI ranged from 0.07 to 0.87 (mean ± SD: 0.28 ± 0.09) (Appendix: Table A1). A summary of results by reproductive mode (Table A1; Figure 6) by population (Table 1) and by clutch (Table A2 and Figure A3) is presented.

Results from the linear mixed‐effects model with heterogeneous residual variances across maternal reproductive strategies (*model.1*) indicated that offspring body condition was significantly associated with clutch size (*nclutch_scaled*), with larger clutches linked to lower individual condition (*p* = 0.002). A quadratic term for maternal size (*fsvl_scaled_sq*) was also significant (*p* = 0.039), indicating a non‐linear relationship between maternal size and offspring condition. Maternal reproductive mode (*fem_mode*) had a significant influence on offspring condition. Post hoc comparisons revealed that offspring from mixed clutches had significantly higher body condition than those from larviparous clutches (*p* = 0.026). Offspring from pueriparous clutches did not differ significantly from either larviparous (*p* = 0.242) or mixed‐strategy clutches (*p* = 0.838). In contrast, individual character stage (*offspring_stage*) had no significant effect on body condition, and no pairwise differences were detected among larviparous, mixed, or pueriparous individuals (all *p* > 0.13) (Table A3).

Random effects analysis indicated that both female identity and population contributed to substantial variance in offspring body condition. The estimated variance components were 0.009 for female identity (SD = 0.095) and 0.008 for population (SD = 0.088), with a residual variance of 0.043 (SD = 0.208) (Table A3). Residual variance differed across maternal reproductive modes, with standard deviations estimated at 1.10 for larviparous females, 1.00 for mixed mode females (reference group), and 0.65 for pueriparous females. The adjusted intraclass correlation coefficient (ICC) was 0.265, indicating that approximately 27% of the variation in offspring condition was attributable to grouping factors (female and population). The marginal R^2^ (fixed effects only) was 0.297, and the conditional R^2^ (including random effects) was 0.493. Model diagnostics revealed no major assumption violations and supported the inclusion of heterogeneous residual variances across maternal strategies (see Text A1 and Figure A1).

## 4 Discussion

This study reveals that hybridization between two phenotypically divergent and relatively old lineages of *S. salamandra* that differ in their reproductive modes (TMRC: 1.3 Mya; Velo‐Antón et al. 2021) results in mixed reproductive modes and intermediate forms along a hybrid cline. We provide insights into life‐history trade‐offs across reproductive strategies within this hybrid zone and the evolution of pueriparity in this unique reproductively bimodal system.

### 4.1 Reproductive Cline: Geographic Variation in Reproductive Modes

Given the genetic admixture and introgression between larviparous *S. s. gallaica* and pueriparous *S. s. bernardezi*, as well as the mixture of phenotypes typical of both subspecies across this contact zone (Velo‐Antón et al. 2021), we hypothesized a cline on reproductive modes, where the southernmost and northernmost extremes of the cline consist of “pure” reproductive forms, while mixed modes are found in central populations. Our results generally supported our predictions, revealing a cline in reproductive modes across our study area, from pure pueriparous forms in the north to pure larviparous forms in the south, with intermediate populations exhibiting mixed modes. The presence of transitional forms along this continuum, makes this contact zone ideal for studying the evolution of reproductive shifts (Recknagel et al. 2021; Whittington et al. 2022).

Pueriparity in *S. salamandra* is associated with heterochronic modifications in ontogenetic timing, including accelerated and asynchronous development, in which embryos within the same oviduct exhibit markedly different developmental stages (Buckley et al. 2007; Buckley 2012; Alarcón‐Ríos and Velo‐Antón 2024). In contrast, births of larviparous females show homogeneity in developmental stages because all the fertilized eggs develop synchronously (Dopazo and Alberch 1994; Buckley et al. 2007), although minor variation in development rate has been reported (Sanchez et al. 2018). In pueriparous females, however, early hatching occurs within the maternal oviducts, and asynchronous development is linked to cannibalistic behavior, such as intra‐oviductal ingestion of eggs (oophagy) or siblings (adelphophagy). We observed cannibalistic behavior in one mixed clutch obtained from Callou (CAL_05), where one larva was seen eating an aborted egg (data not shown). In the same clutch, all individuals showed pigmentation stage LP and aquatic gill morphology (L1), consistent with the onset of heterochronic changes. These developmental shifts at the individual level first appear in Callou and become more frequent toward the north. In the centre of the cline, we observed a shallow transition over approximately 50 km, from larviparity to pueriparity, suggesting little to no selection against hybrids (Abbott et al. 2016; Butlin and Smadja 2018; Nosil et al. 2017). Consistent with the hypothesized gradual shift in reproductive modes, we also found a gradual increase in the proportion of pueriparous individuals toward the north, paralleled by a decrease in larviparous individuals. Mixed phenotypes peaked in central populations but declined further north as pueriparity became predominant. This pattern reflects a gradual, continuous shift in reproductive modes across the contact zone, where hybrids appear to be viable and fertile. Parity mode in *S. salamandra* is an adaptive, genetically determined trait rather than a plastic response (Mulder et al. 2022; Dinis et al. 2025). However, the behavioral dimension of these reproductive strategies remains poorly understood, particularly in mixed populations where parturition site choice may influence offspring performance. A more detailed behavioral assessment—such as continuous video monitoring—would provide valuable insights into birth site selection and parturition behavior in mixed populations.

Intraspecific hybridization and subsequent backcrossing between parity modes have been studied in other squamate models such as *Z. vivipara*, where hybrids exhibit intermediate developmental traits in eggshell thickness and embryonic development (Arrayago et al. 1996; Recknagel et al. 2021). Despite the presence of admixed individuals, reproductive isolation between oviparous and viviparous populations is common in this system, characterized by steep reproductive (< 1 km) transitions and reduced hybrid fitness—suggesting strong selection against hybrids (Lindtke et al. 2010; Cornetti, Belluardo, et al. 2015; Cornetti, Ficetola, et al. 2015; Horreo et al. 2019). Genes linked to parity mode are under divergent and directional selection, likely due to their role in controlling key reproductive traits (Recknagel et al. 2021), which may accelerate viviparity evolution and explain the scarcity of intermediate forms in nature (Horreo et al. 2019; Recknagel et al. 2021). However, our study reveals a broad and permeable hybrid zone between larviparous and pueriparous forms in *S. salamandra* . Here, the gradient of reproductive modes is mirrored by genetic variation (Velo‐Antón et al. 2021) and the contact zone consists mainly of introgressed individuals with varying levels of admixture—indicative of extensive gene flow and recombination. Unlike *Zootoca*, this contact zone does not appear to be maintained by strong genome‐wide selection against hybrids (Figueiredo‐Vázquez et al., in prep.), and we find no evidence of pre‐ or postzygotic barriers among hybrids (Velo‐Antón et al. 2021). Our study highlights a more gradual and continuous reproductive transition, with viable and frequent intermediate phenotypes.

We acknowledge the limited sample size for pueriparous populations. During our fieldwork campaigns between 2022 and 2024, we attempted to collect data on births from four potentially pueriparous populations, but we only managed to record one birth, even though several captured females were clearly gravid (Table 1). We also conducted consistent, albeit opportunistic, searches for larvae in waterbodies during this period (see also Galán 2007), without success. The scarcity of clutch data from the northernmost populations likely reflects the difficulty of obtaining reliable reproductive data in the field or in semi‐natural conditions (i.e., without captive breeding), rather than an actual absence of reproductive activity. Nevertheless, the small number of births restricts our capacity to fully characterize variation within pueriparous populations and to determine whether the observed patterns would persist with increased sampling. Additional sampling across northern populations may reveal greater variability within pueriparous births—for example, a higher frequency of P1_P pueriparous individuals toward the south. These challenges highlight the logistical difficulties involved in obtaining reproductive data from natural populations. Future efforts to integrate long‐term, systematic monitoring across replicated transects would help to overcome these sampling limitations and provide a more comprehensive understanding of spatial variation in reproductive strategies.

### 4.2 Variation in Offspring Body Condition—Differences Between Reproductive Modes

This system poses a valuable opportunity to study life‐history trait variation while minimizing the confounding interferences of environmental and phylogenetic factors (Dunham and Miles 1985; Meiri et al. 2012; Bassar et al. 2014), allowing for the examination of life‐history variation between the two recently diverged reproductive modes (Blackburn 2006; Murphy and Thompson 2011; Velo‐Antón et al. 2015; Mulder et al. 2022). Because reproductive modes in *S. salamandra* differ in maternal investment through matrotrophy, variation in offspring body condition (SMI) provides an informative proxy for life‐history variation across reproductive modes. The existence of matrotrophy (i.e., oophagy and adelphophagy) in pueriparous and mixed females may allow offspring to obtain a higher body condition than those larviparous females in which their offspring solely rely on lecithotrophic reserves, and thus compensating for reduced fecundity (Kupfer et al. 2006; Velo‐Antón et al. 2015). Our results showed that maternal reproductive mode (*fem_mode*) significantly influenced offspring condition. Offspring from mixed clutches (i.e., females) had the highest SMI values—significantly higher than those of larviparous clutches and, although not significantly, those of pueriparous clutches. Clutch size, was negatively associated with offspring condition, supporting the classic trade‐off between offspring number and quality (Stearns 1976; Sinervo 1990), where smaller clutches may enhance offspring survival or independence. Surprisingly, at the individual level, offspring developmental character stage (*offspring_stage*) had no significant effect on body condition.

We predict that the elevated SMI of offspring from mixed clutches may result from asymmetries in intra‐brood competitive abilities for maternal resources. In mixed clutches, pueriparous embryos may benefit from sharing the uterus with lecithotrophic larviparous siblings, that do not compete for matrotrophic inputs, allowing greater resource allocation to matrotrophic individuals. This suggests that the interaction between reproductive mode and clutch composition—that is, the intrauterine context in which offspring develop, defined by sibling number and reproductive mode composition—may shape offspring body condition more than clutch parity mode alone or offspring developmental stage. Notably, offspring from pueriparous clutches did not differ significantly in body condition from offspring from either larviparous or mixed clutches, indicating no consistent advantage (or disadvantage) of this reproductive mode, at least in terms of body condition in the sampled populations. However, these offspring showed the lowest residual variance in body condition, which may result from postcopulatory processes such as increased sibling competition within the female (i.e., within a limited space). This could selectively favor higher‐performing individuals, effectively filtering out offspring with lower body condition and leading to more homogeneous clutches (Alarcón‐Ríos and Velo‐Antón 2024).

Previous studies in *S. salamandra* have shown that mass and total size of the larvae decreased with their date of deposition (Caspers et al. 2014; Alarcón‐Ríos and Velo‐Antón 2024). As a result, earlier‐born larvae may exhibit better condition. Conversely, in pueriparous females, where embryos hatch early within the uterus and move freely within it, birth order may be decoupled from oviductal position (Alarcón‐Ríos and Velo‐Antón 2024), and active feeding behavior within the uterus of those later‐hatching and smaller individuals could result in an increase of total mass regarding their size, and thus their body condition at birth. Hence, this pattern is consistent with our residual variance estimates, where larviparous and pueriparous clutches show the highest and lowest variance in body condition, respectively.

Maternal size showed no linear relationship with offspring body condition; however, a weak but significant non‐linear effect was detected, potentially reflecting size‐dependent reproductive trade‐offs. This pattern might suggest that both smaller and larger females produce fewer offspring in better condition, potentially reflecting different trade‐offs between offspring number and quality.

While our findings suggest potential advantages for mixed reproductive modes resulting from hybridization, these hypotheses remain speculative due to several limitations. First, we lack data on the number and size of ovulated and fertilized eggs per female (Alcobendas et al. 2004; Buckley et al. 2007; Dopazo and Alberch 1994; Dopazo and Korenblum 2000), which is essential to understanding how many eggs are reallocated toward matrotrophy—a key factor influencing resource distribution and offspring condition. Additionally, differences in mating strategies (monandry vs. polyandry, Alarcón‐Ríos and Velo‐Antón 2024), intrinsic genetic factors associated with larval birth size (Travis et al. 1987; Dopazo and Alberch 1994; Semlitsch and Schmiedehausen 1994; Veith et al. 1998; Alcobendas et al. 2004; Caspers et al. 2015), and environmental variability (Alcobendas et al. 2004; Caspers et al. 2015; Eclair and Rebelo 2003; Kaplan and Cooper 1984) may also contribute to differences in offspring traits. Moreover, stress experienced by females during captivity could have influenced developmental outcomes and offspring body condition. Although all females were maintained under the same outdoor enclosure conditions to minimize such effects, variation in gestational stage at capture may have introduced confounding effects. Females sampled at later stages of pregnancy might be more susceptible to stress‐induced premature birth, while those captured earlier may have experienced different pregnancy conditions during captivity, potentially introducing additional variability in developmental outcomes. We cannot fully rule out the possibility that an incomplete acclimatization to enclosure conditions might prevent the obtention of further births; nevertheless, the same conditions were used in previous studies, providing a high rate of successful parturitions (Velo‐Antón et al. 2015; Alarcón‐Ríos, Nicieza, Lourenço, and Velo‐Antón 2020; Mulder et al. 2022; Alarcón‐Ríos and Velo‐Antón 2024). Moreover, opportunistic field observations suggest a similar latitudinal pattern in reproductive mode as seen in captivity, supporting our findings.

Notably, random effects associated with population and female identity explained a substantial portion of the variance in offspring condition. This may partially reflect the limited sample size, which can reduce statistical power. Future research would benefit from increased sampling and experimental approaches, including tests of hybrid performance and survival, ideally within an integrative framework combining in vitro analyses of embryonic development across reproductive modes. Such work could clarify whether mixed reproductive modes confer selective advantages through enhanced access to maternal resources—potentially via mechanisms such as accelerated growth or competitive embryonic behavior.

## 5 Conclusions

Reproductive traits influence both microevolutionary processes (e.g., dispersal/gene flow: Lourenço et al. 2019; Figueiredo‐Vázquez et al. 2021; increased parental care: Gomez‐Mestre et al. 2012; Vági et al. 2019) and macroevolutionary events, such as habitat colonization (Velo‐Antón et al. 2007; Lourenço et al. 2017; Domínguez‐Guerrero et al. 2024) and lineage diversification (Helmstetter et al. 2016; Liedtke et al. 2022). Transitional forms must therefore offer some selective advantage to be maintained over time. In bimodal species, transitional phenotypes are not common but may persist if they are more advantageous than alternative reproductive strategies under certain environmental conditions (Whittington et al. 2022).

There is likely an evolutionary advantage to salamanders giving birth to both larvae and metamorphosed juveniles, or to mixed individuals, under changing and unpredictable environmental conditions. In our study, offspring from mixed clutches showed higher body condition, which may help reduce predation risk in aquatic environments by allowing them to bypass early larval stages. Moreover, mixed‐mode parturition may be advantageous in habitats where suitable aquatic sites are scarce or unpredictable. By producing at least some terrestrial or mixed offspring, females may increase the chances of brood survival—a strategy that could become increasingly important with rising drought frequency and associated hydric stress (Russo et al. 2015). These ecological benefits likely contribute to the survival of fire salamander populations across the contact zone and suggest that genetic factors and novel trait combinations arising from hybridization and introgression may facilitate their persistence in the long term. Hybridization can generate new combinations that offer advantages under environmental change and may help overcome developmental constraints associated with intermediate forms. Yet, we could not directly measure female reproductive investment, and thus we cannot assess the cost or burden of these intermediate forms (Whittington et al. 2022). Evaluating such costs is an important next step for understanding the fitness consequences of transitional reproductive modes.

## Author Contributions

**Guillermo Velo‐Antón:** conceptualization (lead), data curation (equal), formal analysis (supporting), investigation (equal), supervision (lead), writing – review and editing (equal). **Lucía Alarcón‐Ríos:** conceptualization (equal), data curation (equal), formal analysis (equal), investigation (equal), supervision (equal), writing – review and editing (equal). **Clara Figueiredo‐Vázquez:** conceptualization (equal), data curation (lead), formal analysis (lead), investigation (lead), writing – original draft (lead), writing – review and editing (equal).

## Funding

This work was supported by Ramón y Cajal research grant, RYC‐2019‐026959‐I/AEI/https://doi.org/10.13039/501100011033; Fundação para a Ciência e a Tecnologia, 2021.07890.BD.

## Conflicts of Interest

The authors declare no conflicts of interest.

## Data Availability

Data and code have been deposited in Figshare (DOI: https://doi.org/10.6084/m9.figshare.29900897).

## Appendix

**TABLE A1:** Descriptive statistics for SMI, total length and weight across three hierarchical classification levels. At the offspring level, categories correspond to developmental character stages (larval, juvenile, mixed).

Variable	Category	Offspring stage			Rep. mode clutch			Population composition		
		Mean ± SD	Median	Range	Mean ± SD	Median	Range	Mean ± SD	Median	Range
SMI	Pueriparous	0.295 ± 0.059	0.293	0.196–0.587	0.278 ± 0.047	0.275	0.196–0.390	0.241 ± 0.023	0.236	0.196–0.277
	Mixed	0.319 ± 0.104	0.287	0.195–0.661	0.298 ± 0.098	0.284	0.132–0.869	0.290 ± 0.089	0.269	0.092–0.869
	Larviparous	0.264 ± 0.082	0.250	0.065–0.869	0.253 ± 0.067	0.245	0.067–0.609	0.236 ± 0.060	0.236	0.065–0.602

## Text A1 Model validation

We assessed model assumptions using the check_model() function from the performance package (Lüdecke et al. 2021), which provides graphical diagnostics of linearity, collinearity, and residual distribution. These diagnostics indicated good overall model fit, low collinearity, and residuals approximately normally distributed, with only slight deviations from homoscedasticity at the extremes of fitted values (Figure A1).

To test whether allowing for heterogeneity in variance across maternal reproductive strategies improved model fit, we fitted a homoscedastic linear mixed‐effects model (*model.2_homo*) with the same fixed and random structure as *model.1*, but assuming constant residual variance, using the lmer() function from the lme4 package (Bates et al. 2015).

We validated *model.2_homo* using DHARMa residual diagnostics (Hartig et al. 2024). The Kolmogorov–Smirnov test indicated no deviation from the expected uniform distribution of residuals (KS test: *P* = 0.289), and no evidence of overdispersion was found (dispersion test: *p* = 0.816). The DHARMa bootstrap outlier test detected 8 marginal observations out of 465 (expected frequency = 0.0123; observed = 0.0172), but this did not differ significantly from the expected number under the model (*p* = 0.48). However, residual versus predictor plots showed patterns of deviation for *fsvl_scaled_sq* and fitted values, and the combined quantile test was significant, suggesting residual structure not fully captured by the model.

The likelihood ratio test supported the heteroscedastic *model.1* over *model.2_homo* (*χ* ^2^ = 6.76, df = 2, *p* = 0.034), which also had a lower AIC (−4.40 vs. –1.63) and higher log‐likelihood (15.20 vs. 11.82). Given their identical fixed and random structures and similar results, but superior fit for *model.1*, we selected it as the preferred model. These results indicate that accounting for heterogeneous residual variances across maternal reproductive modes significantly improves model performance and supports the use of *model.1*.

## Appendix

**TABLE A2:** Sampled populations and corresponding female codes. For each female, the number of newborns (*N*), larvae (Lar.), juveniles (Juv.), and mixed individuals is reported. The mean, standard deviation, and median are provided for total length (TL), weight, and Scaled Mass Index (SMI) of each clutch.

Population	Female	*N*	Lar.	Juv.	Mixed	TL (mm)		Weight (g)		SMI	
						Mean ± SD	Median	Mean ± SD	Median	Mean ± SD	Median
CARIÑO	CAR_41F	12	0	12	0	35.81 ± 3.6	35.77	0.33 ± 0.11	0.3	0.24 ± 0.02	0.24
VICEDO	VIC_01F	9	0	9	0	NA	NA	NA	NA	NA	NA
CERVO	CER_26F	5	3	1	1	26.40 ± 3.48	28.25	0.19 ± 0.05	0.2	0.48 ± 0.22	0.38
	CER_27F	23	9	8	6	28.16 ± 2.74	27.81	0.20 ± 0.09	0.18	0.35 ± 0.07	0.35
CEDEIRA	CED_33F	5	0	5	0	32.41 ± 2.14	31.44	0.28 ± 0.05	0.27	0.32 ± 0.04	0.3
	CED_34F	9^a^	6	0	2	29.8 ± 3.37	31	0.27 ± 0.07	0.28	0.41 ± 0.13	0.35
VALDOVIÑO	VAL_12F	8	0	8	0	35.48 ± 2.75	34.89	0.40 ± 0.16	0.36	0.30 ± 0.03	0.3
	VAL_16F	7^a^	1	5	0	42.36 ± 3.92	43.44	0.64 ± 0.23	0.65	0.25 ± 0.03	0.24
	VAL_30F	16	3	11	2	33.32 ± 1.48	33.33	0.32 ± 0.07	0.31	0.31 ± 0.06	0.3
	VAL_36F	15^a^	7	7	0	31.47 ± 1.92	31.02	0.25 ± 0.05	0.24	0.30 ± 0.03	0.3
BELELLE	BEL_20F	19^a^	12	NA	NA	35.26 ± 3.12	35.79	0.33 ± 0.07	0.34	0.28 ± 0.11	0.23
	BEL_21F	3	0	3	0	41.01 ± 3.29	40.26	0.64 ± 0.09	0.66	0.30 ± 0.08	0.26
EUME	EUM_30F	9	6	0	3	37.77 ± 4.64	39.42	0.52 ± 0.16	0.5	0.33 ± 0.11	0.28
	EUM_31F	11	3	7	1	37.59 ± 5.52	39.6	0.50 ± 0.14	0.51	0.33 ± 0.12	0.29
	EUM_32F	37^a^	28	0	3	32.26 ± 1.76	32.11	0.20 ± 0.06	0.17	0.22 ± 0.05	0.22
BAXOI	BAX_36F	15^a^	8	2	4	37.88 ± 3.63	37.7	0.44 ± 0.2	0.39	0.25 ± 0.03	0.26
	BAX_37F	18^a^	12	0	2	34.57 ± 3.28	35.25	0.33 ± 0.09	0.33	0.28 ± 0.04	0.29
	BAX_38F	37^a^	30	0	3	31.51 ± 2.66	31.22	0.19 ± 0.05	0.17	0.23 ± 0.04	0.22
	BAX_39F	10^a^	5	0	0	41.75 ± 4.52	41.49	0.57 ± 0.18	0.56	0.24 ± 0.05	0.23
LAMBRE	LAM_39F	20^a^	12	0	3	37.52 ± 2.85	37.31	0.39 ± 0.14	0.38	0.36 ± 0.08	0.34
	LAM_41F	9^a^	1	0	3	37.44 ± 1.62	37.05	0.72 ± 0.22	0.73	0.47 ± 0.19	0.49
	LAM_43F	16	7	1	8	34.14 ± 4.11	33.57	0.46 ± 0.08	0.46	0.30 ± 0.09	0.27
	LAM_44F	8^a^	2	5	0	39.09 ± 3.43	39.42	0.53 ± 0.14	0.59	0.28 ± 0.04	0.3
CALLOU	CAL_01F	21	21	0	0	34.21 ± 2.81	34.29	0.33 ± 0.07	0.34	0.30 ± 0.08	0.31
	CAL_02F	33	33	0	0	30.45 ± 1.35	30.21	0.18 ± 0.03	0.19	0.26 ± 0.04	0.26
	CAL_03F	38^a^	37	0	0	33.77 ± 2.67	33.34	0.26 ± 0.03	0.26	0.26 ± 0.06	0.26
	CAL_05F	5^a^	0	0	4	42.74 ± 1.3	43.36	1.01 ± 0.16	1.02	0.4 ± 0.06	0.42
CESURAS	CES_01F	25	25	0	0	30.92 ± 2.6	30.36	0.17 ± 0.05	0.15	0.22 ± 0.03	0.21
	CES_03F	43^a^	34	0	0	28.97 ± 0.83	29.01	0.16 ± 0.03	0.15	0.26 ± 0.05	0.26
CHAIAN	CHA_01F	23^a^	21	0	0	33.46 ± 3.24	33.65	0.25 ± 0.06	0.24	0.25 ± 0.09	0.23
	CHA_03F	7	7	0	0	29.26 ± 1.75	27.81	0.14 ± 0.01	0.14	0.24 ± 0.07	0.21
	CHA_05F	32^a^	29	0	0	31.8 ± 3.09	31.12	0.17 ± 0.03	0.17	0.20 ± 0.06	0.22

^a^ For some offspring from these individuals, it was not possible to determine the developmental stage of gills and/or pigmentation because they were either highly degraded at the time of processing or represented embryos at advanced developmental stages.

**TABLE A3:** Summary of the linear mixed‐effects model with heterogeneous residual variances (model.1), including fixed effect estimates (*β*) with corresponding *p*‐values (*p*), random effect variances (Var.) and Standard Deviations (SD).

Model	Fixed effect statistics																Random effect statistics					
	Intercept (*β*)		fsvl_scaled		fsvl_scaled_sq		nclutch_scaled		offspring_stage_mixed		offspring_stage_pueriparous		fem_modemixed		fem_modepueriparous		Female: pop		Pop		Residual	
	*β*	*p*	*β*	*p*	*β*	*p*	*β*	*p*	*β*	*p*	*β*	*p*	*β*	*p*	*β*	*p*	Var.	SD	Var.	SD	Var.	SD
*model.1*	−1.461	0.000	0.003	0.934	0.039	0.039	−0.101	0.00	−0.033	0.400	−0.067	0.136	0.175	0.026	0.129	0.242	0.009	0.095	0.008	0.088	0.043	0.208
